# A Study on the Relationship between Food Security and the Number of Remaining Teeth in Korean Adults: The Korea National Health and Nutrition Examination Survey (KNHANES VII), 2016–2018

**DOI:** 10.3390/ijerph20042964

**Published:** 2023-02-08

**Authors:** Su-Yeon Hwang, Jung-Eun Park

**Affiliations:** 1 Department of Dental Hygiene, Daejeon Institute of Science and Technology, Daejeon 35408, Republic of Korea; 2 Department of Dental Hygiene, College of Health Science, Dankook University, Cheonan-si 31116, Republic of Korea

**Keywords:** food security, dental caries, nutrition surveys, oral health, periodontal disease

## Abstract

Food security is important for human health and quality of life. This study investigated the association between food security and the number of remaining teeth in Korean adults. Raw data from the Korea National Health and Nutrition Examination Survey (KNHANES) VII (2016–2018) were analyzed, including data from 13,199 adults aged 19 years or older. The associations between food security and number of teeth were assessed using multiple multinomial logistic regression models after adjusting for demographic and health factors as covariates. In the model adjusted for all socioeconomic, medical, and behavior variables, the odds ratio of tooth loss (16–20 teeth) was 3.80 (95% confidence interval [CI] 1.56–9.21) in the group of people that reported often feeling insecurity regarding various food groups compared to those who reported feeling food security. The results of this study demonstrated an association between food security and the number of remaining teeth in Korean adults. Therefore, food security is essential for improving lifelong oral health.

## 1. Introduction

Tooth extraction is the final approach when prophylactic and conservative treatments for oral diseases are no longer possible [1]. The resulting tooth loss is considered a lifetime cumulative indicator of poor oral health [2].

The most common oral diseases associated with tooth loss are periodontal disease and dental caries. Periodontal disease is caused by bacterial accumulation on the surface of the teeth and dysregulation of local and systemic immune responses. The damage to connective tissue and loss of alveolar bone around the teeth can lead to tooth loss at the terminal stage [3,4]. Dental caries is caused by acid produced through the bacterial fermentation of dietary carbohydrates. Subsequently, hard dental tissues are demineralized by acid, causing dental caries [5]. This can progress from early dental caries to severe tooth destruction, eventually leading to tooth loss. Tooth loss is caused not only by biological but also by socio-economic and health-related behavioral factors [6,7,8]. Therefore, oral disease can result from not only the effects of biofilm on tooth surfaces but also from complex factors.

Studies have reported associations between nutrition and oral health. A review of nutrition and oral health suggested that malnutrition might negatively affect orofacial components, oral mucosa, and dental diseases [9]. In addition, a study on the association between the number of teeth and dietary quality (Healthy Eating Index) reported a positive association between the number of teeth and dietary quality [10]. Hence, nutrition and diet-related factors can significantly impact both oral and overall health. Nutritional intake is affected by household access to food and the environment. Therefore, nutritional intake should be assessed according to food security.

Food security includes household access to and the sufficiency and sustainability of food to meet the dietary needs of all household members [11]. Food insecurity is a risk factor for non-infectious diseases and is also a physiological and psychological stressor through various pathways [12]. In other words, food security does not simply mean that people do not eat enough food. Food security is also an indicator predicting the socioeconomic inequality and health of a population [13]. Thus, food insecurity can contribute to the development of dental caries due to the consumption of low-quality foods and even higher levels of free sugars. [14].

Recent studies have reported on the relationships between food security and dental diseases. A study of Korean adults observed that food insecurity was associated with an increased prevalence of dental caries [15]. In addition, a study involving schoolchildren in western Brazil also reported a close association between food insecurity and dental caries [16].

However, to our knowledge, no studies have evaluated the association between food security and the number of remaining teeth. The number of remaining teeth can be used as an indicator of the overall oral health status, including dental caries and periodontal disease. In addition, multifaceted approaches and measures for oral health can be developed through studies on the number of remaining teeth and food security. Therefore, the present study investigated the association between dietary life conditions related to food security and the number of remaining teeth in Korean adults based on an analysis of nationally representative data in South Korea.

## 2. Materials and Methods

### 2.1. Study Participants

This study used the data from the 7th Korea National Health and Nutrition Examination Survey (KNHANES), a nationally representative cross-sectional survey conducted from 2016 to 2018. The data from the KNHANES VII Survey can be accessed and downloaded from the KNHANES homepage (URL: https://knhanes.kdca.go.kr/knhanes/sub03/sub03_02_05.do, accessed on 21 November 2022). The first and second years of the 7th KNHANES (2016–2017) collected data without requiring an institutional review board (IRB) review and approval according to the Bioethics Act, while the third year (2018) collected data after receiving approval from the IRB at the Korea Disease Control and Prevention Agency (IRB No. 2018-01-03-P-A). Among 16,489 survey participants, data from 13,199 participants aged 19 years or older were extracted.

### 2.2. Number of Teeth

In the KNHANES, standardized oral examinations have been continuously performed by highly trained dentists. The methods used to ensure the reliability of the oral examinations by dentists include lectures for calibration, training using dental models, web-based photo instruction and simulation of oral health examination with human subjects, field instruction, and reproducing examinations.

The number of remaining teeth as a dependent variable was calculated after excluding the third molar. The number of remaining teeth of participants was categorized into three groups: ≤15 teeth, 16–20 teeth, and 21–28 teeth according to the classification used in previous studies [17,18].

### 2.3. Food Security

Dietary life condition as an independent variable was analyzed using dietary condition data from the food security survey of the 7th KNHANES. Each household diet manager was surveyed. The participants were asked to select response items that indicated their dietary life conditions in the past year, according to their overall household situation: (1) All my family members ate sufficient quantities of various kinds of food as they want (1 = secure with various food), (2) my family members ate a sufficient amount of food but could not eat various types of food (2 = secure without various food), (3) my family sometimes lacked food due to financial difficulties. (3 = sometimes insecure), and (4) my family often lacked food because of financial difficulties (4 = often insecure). Based on these four response items, the participants’ dietary life condition was categorized.

### 2.4. Covariates

Each variable in this study was reclassified according to its analysis purpose. The demographic factors included sex, age, household income level, and education level. Age was categorized into 19–29, 30–39, 40–49, 50–59, and 60 years of age or older. Household income was categorized into upper, middle (upper-middle, lower-middle), and lower. Education level was categorized into primary school graduation or lower, middle school graduation, high school graduation, and college graduation or higher. Regarding health factors, diabetes was classified as a fasting blood glucose level ≥ 126 mg/dL, a diabetes diagnosis by a doctor, or taking a drug for glycemic management or receiving insulin injections [19]. Body mass index (BMI) was divided into underweight (<18.5 kg/m^2^), normal weight (18.5–22.9 kg/m^2^), overweight (23–24.9 kg/m^2^), and obese (>25 kg/m^2^).

Regarding health-related behavioral factors, smoking status was divided into non-smokers (<5 packs in their lifetime) and smokers (>5 packs in their lifetime) [20,21]. Drinking status was classified according to monthly drinking rate into non-drinkers (non-drinkers in their lifetime or <1 drink per month in the past year) and drinkers (>1 drink per month in the past year) [22]. The number of tooth brushings per day was divided into ≤1, 2, and ≥3.

### 2.5. Statistical Analysis

The KNHANES used a complex sample design to collect data, and a complex sample analysis of the collected data was performed after applying weights, stratification variables (kstrata), and primary sample units (psu).

The number of teeth according to the general characteristics of the study participants was analyzed using a Chi^2^ test. The number of teeth according to the general characteristics was analyzed using *t*-tests and analysis of variance (ANOVA). Multiple multinominal logistic regression models were used to determine the number of teeth according to food security. Model 1 was unadjusted, while Model 2 was adjusted for sex, age, household income, education level, diabetes, BMI, smoking, drinking, and number of tooth brushings per day. The results of the analysis were expressed as the odds ratio of the number of teeth and 95% confidence intervals (CIs). All statistical analyses in this study were performed using IBM SPSS Statistics for Windows, Version 20.0 (IBM Corp., Armonk, NY, USA). The tests of significance were based on a type I error level of 0.05.

## 3. Results

### 3.1. Categories of the Number of Teeth According to the General Characteristics of the Participants

The categories of the numbers of teeth according to the general participant characteristics are shown in Table 1. Female sex, higher age, lower household income level, and lower education level were associated with an increased proportion of participants with ≤15 teeth (*p* < 0.001). In addition, the proportion of participants with ≤15 teeth was higher in those without diabetes (77.4%, *p* < 0.001). The proportion of participants with ≤15 teeth was high (36.2%) among those with normal weight and those with obesity (*p* < 0.001). The proportion of participants with ≤15 teeth was also in non-smokers and non-drinkers (80.7%, *p* = 0.198 and 62.9%, *p* < 0.001, respectively). The proportion of participants who brushed their teeth two times per day was also high (44.3%, *p* < 0.001).

### 3.2. Number of Teeth According to General Characteristics

The mean numbers of teeth according to the general participant characteristics are shown in Table 2. The number of teeth was 0.9 higher in women than in men. The number of teeth decreased with increasing age (*p* < 0.001). Higher household income and education level were associated with greater numbers of teeth (*p* < 0.001). The number of teeth was lower in participants with diabetes (*p* < 0.001), those who were underweight (*p* = 0.011), smokers (*p* = 0.016), and non-drinkers (*p* < 0.001). In addition, the number of remaining teeth was higher with increasing numbers of tooth brushings per day (*p* < 0.001). Finally, while the number of remaining teeth was the lowest (22.68) in those who reported that they are food insecure and often lack food; however, the difference was not statistically significant (*p* = 0.378).

### 3.3. Food Security According to General Characteristics

Food security according to the general characteristics is shown in Table 3. Females (58.7%) had better dietary habits than males (41.3%, *p* < 0.001), and those aged 60 or older (32.5%) reported good food security (*p* < 0.001). The most common level of household income was median (50.2%), followed by high (35.5%) and low (14.3%, *p* < 0.001). Food security increased with the level of education (*p* < 0.001), from primary school graduate or lower (16.1%), middle school graduate or lower (8.7%), high school graduate or lower (32.2%), to university graduate or higher (43.4%). Non-diabetics (*p* < 0.001), those with normal BMI (*p* = 0.211), non-smokers (*p* < 0.001), drinkers (*p* < 0.001), and those who brush their teeth more than three times a day (*p* < 0.001) reported having good food security.

### 3.4. Associations between Food Security and the Number of Teeth

The associations between food security and the number of teeth are shown in Table 4. In Model 1, with 0–15 teeth, the odds ratios of tooth loss in the groups of participants who reported experiencing food insecurity often, sometimes, and only for certain food groups compared to those without food insecurity were 5.52, 3.78, and 1.38, respectively. In Model 2, adjusted for all variables, the odds ratios were not significant.

In Model 1, with 16–20 teeth, the odds ratios of tooth loss in the groups of participants who reported experiencing food insecurity often, sometimes, and only for certain food groups compared to those without food insecurity were 5.36, 2.85, and 1.24, respectively. In Model 2, the odds ratio of tooth loss in those who often experienced food insecurity was 3.80 (1.56–9.21) compared to those without food insecurity.

## 4. Discussion

This study analyzed the association between food security and the number of remaining teeth using raw data from the 7th KNHANES, a nationally representative survey of Korean adults.

The results of this study reveal that in the model adjusted for socioeconomic, medical, and behavioral factors, the risk of tooth loss (16–20 remaining teeth) was 3.80-fold higher (95% CI: 1.56–9.21) in the group of participants often experiencing food insecurity for various food groups compared to the group with security. Therefore, severe food insecurity was associated with tooth loss.

Food-related social and cultural norms affect individuals’ food choices, preferences, beliefs, and behaviors [23]. The factors affecting individuals’ food choices are diverse. The main cause of food insecurity in the present study was economic factors. Food prices significantly affected food choices and purchases. Accordingly, households with food insecurity make purchasing decisions based on food quantity rather than quality. This may result in the consumption of refined, high-calorie, low-nutrient, and inexpensive food with a long shelf life [24]. These foods may eventually affect overall nutritional status, such as increased weight, reduced healthy mealtimes, and barriers to access fruits and vegetables in parents with food insecurity [25]. In the long run, this situation can lead to nutritional imbalances and adversely affect both overall and oral health.

The study was limited in identifying a causal relationship between food insecurity and oral health status. A study on health imbalances and food insecurity in Canada reported that diet quality-related food insecurity, such as high sugar intake, limited intake of dairy products, and a low-variety diet, caused poor oral health [26]. Refined carbohydrates directly affect oral health. The intake of highly refined carbohydrates supports the cariogenic bacteria and biofilm growth in the oral cavity [27,28], and has other negative effects such as an increased risk of opportunistic infections and diabetes [29,30].

Moreover, insufficient nutritional intake causes a deterioration of the immune system and affects phagocytic function, cytokine production, and antibody response [31]. Thus, nutrition imbalance and a lack of appropriate nutrition consumption can lead to changes that increase the risk of infection and develop an acidic environment in the mouth. Consequently, this can contribute to inducing oral diseases and tooth loss.

A previous analysis of data of Korean adults from the 6th KNHANES reported a low micronutrient intake and low Healthy Eating Index scores among food-insecure individuals [15]. Micronutrients act as antioxidants and help bones and muscles, with each micronutrient playing diverse roles. Phosphorus, calcium, magnesium, and vitamin D, which affect hard tissues, interact with one another and play roles in the remineralization of bones and teeth [32]. These roles affect the prevention and management of dental caries and periodontal disease, thereby influencing overall oral health.

In addition, a study of adults in the US showed that those with food insecurity had 1.58-fold higher (95% CI: 1.18–2.12) unmet dental needs [24], which are an additional economic burden for food-insecure individuals. These economic burdens can lead to the reduced utilization of healthcare services and the intake of low-quality food. Furthermore, while food-insecure poor persons may visit a dental clinic for dental treatment, their consumption of necessary preventive and therapeutic treatment is limited. Poor persons who recognize the need for dental care may not undergo preventive dental procedures and treatments for promoting oral health due to financial demands. In addition, paying for necessary dental treatment may require them to not buy food or to forcibly reduce and manage food purchase expenses [26].

Furthermore, long delays in necessary dental treatment can lead to future tooth loss and cause oral health problems, which can become a vicious cycle. The loss of teeth greatly affects diet quality. Poor oral condition and chewing discomfort affect an individual’s food choices and dietary preferences. Consequently, the intake of low-quality food has negative effects on overall and oral health [33].

The long-standing accommodative monetary policies and liquidity during the coronavirus disease 2019 (COVID-19) pandemic and the situation in individual countries have led to rapid inflation. Thus, people from lower socioeconomic classes are more likely to be exposed to food insecurity. This is an important issue for the healthy life of people worldwide; thus, policy considerations are needed. The providers and educators of oral health promotion programs should be well aware of the effects of the nutritional status of the subjects on their oral health. Accordingly, the program should be implemented for oral health education and counseling to promote the oral health of the subjects. In addition, measures to connect subjects suffering from severe food instability during medical treatment to related institutions are required.

This study has several limitations. First, owing to the cross-sectional design, the causal relationships could not be determined. Second, a tool to measure food security in Korea based on the US Household Food Security Survey Module was developed in 2011 and used in the 6th KNHANES [34]. However, because this tool consists of rotating survey items, the food security survey was based on a single questionnaire item regarding dietary life condition in the 7th KNHANES (2016–2018). Therefore, it was difficult to accurately measure food security levels using a single-item measurement indicator. Third, multicollinearity could not be assessed in this study because a complex sample analysis was performed.

Nevertheless, to our knowledge, this is the first study to determine the association between food security and the number of remaining teeth as a cumulative indicator of oral diseases in Korean adults. Longitudinal studies are needed to analyze changing trends in food security and the number of teeth based on the measurement tool for food security in Korea.

## 5. Conclusions

The group of participants who reported being often insecure (the worst food security category) showed an association with the number of remaining teeth. Regarding health consultations, oral health professionals should offer patients advice on food security and links to various support resources.

## Figures and Tables

**Table 1:** Characteristics of the study population stratified by number of teeth.

Characteristics		Number of Teeth						*p*-Value **
		0–15		16–20		21–28		
		N	% *	N	% *	N	% *	
Sex (n = 13,199)	Man	774	47.6	341	44.8	4678	41.5	<0.001
	Woman	858	52.4	414	55.2	6134	58.5	
Age (n = 13,199)	19–29	1	0.0	2	0.2	1559	15.1	<0.001
	30–39	4	0.3	11	1.6	2087	18.7	
	40–49	19	1.2	32	4.0	2368	21.1	
	50–59	126	8.2	145	20.2	2222	21.6	
	≥60	1482	90.4	565	74.0	2576	23.6	
House income (n = 13,161)	Lower	872	51.9	285	35.9	1484	13.8	<0.001
	Median	628	39.9	349	47.9	5834	53.4	
	Upper	122	8.1	120	16.2	3467	32.9	
Education	≤Primary school	936	60.4	326	43.0	1367	12.9	<0.001
(n = 12,566)	Middle school	217	15.0	121	17.2	895	8.8	
	High school	253	18.1	167	25.9	3608	35.3	
	≥College	96	6.5	91	13.9	4489	43.0	
Diabetes (n = 13,199)	Absence	1242	77.4	609	81.6	10,042	93.1	<0.001
	Presence	390	22.6	146	18.4	770	6.9	
BMI	Underweight	60	4.2	19	2.4	421	4.0	<0.001
(n = 12,880)	Normal	558	36.2	202	28.2	4161	40.2	
	Overweight	387	23.5	214	28.9	2299	21.9	
	Obesity	565	36.2	297	40.5	3697	33.9	
Smoking (n = 13,061)	Non-smoker	1300	80.7	619	83.3	8795	82.7	0.198
	Smoker	292	19.3	121	16.7	1934	17.3	
Alcohol drinking (n = 13,069)	Non-drinker	1015	62.9	409	54.7	4658	43.6	<0.001
	Drinker	579	37.1	333	45.3	6075	56.4	
Tooth brushing/day	≤1	325	23.0	89	10.6	778	6.8	<0.001
(n = 12,759)	2	619	44.3	313	42.4	4067	38.1	
	≥3	455	32.8	316	47.1	5797	55.0	

* Weighted %; ** *p*–value was calculated by a complex sample chi-square test.

**Table 2:** Mean number of teeth and standard error of the study population.

Characteristics		Number of Teeth	*p*-Value *
		Weighted Mean (SE)	
Sex	Man	21.22 (0.18)	<0.001 ^†^
	Woman	22.12 (0.20)	
Age	19–29	25.15 (0.35)	<0.001
	30–39	24.49 (0.36)	
	40–49	24.48 (0.35)	
	50–59	23.34 (0.35)	
	≥60	19.21 (0.36)	
House income	Lower	22.05 (0.38)	<0.001
	Median	23.88 (0.34)	
	Upper	24.08 (0.35)	
Education	≤Primary school	21.80 (0.42)	<0.001
	Middle school	23.24 (0.40)	
	High school	23.88 (0.34)	
	≥College	24.41 (0.34)	
Diabetes	Absence	23.90 (0.14)	<0.001 ^†^
	Presence	19.43 (0.29)	
BMI	Underweight	22.66 (0.47)	0.011
	Normal	23.43 (0.35)	
	Overweight	23.61 (0.35)	
	Obesity	23.64 (0.34)	
Smoking	Non-smoker	21.93 (0.16)	0.016 ^†^
	Smoker	21.41 (0.24)	
Alcohol drinking	Non-drinker	20.59 (0.21)	<0.001 ^†^
	Drinker	22.75 (0.17)	
Tooth brushing/day	≤1	22.11 (0.44)	<0.001
	2	23.85 (0.33)	
	≥3	24.04 (0.32)	
Food security	F1 = Secure with various food	23.70 (0.16)	0.378
	F2 = Secure without various food	23.84 (0.18)	
	F3 = Sometimes insecure	23.12 (0.58)	
	F4 = Often insecure	22.68 (1.17)	

* *p*-value was calculated by Analysis of Variance (ANOVA) in a complex sampling general linear model. ^†^ *p*-value was calculated by a *t*-test complex sampling general linear model.

**Table 3:** Characteristics of the study population stratified by food security.

Characteristics		Food Security				*p*-Value **
		F1	F2	F3	F4	
		N (%) *	N (%) *	N (%) *	N (%) *	
Sex (n = 11,489)	Man	2612 (41.3)	2069 (40.1)	130 (44.9)	23 (37.5)	<0.307
	Woman	3554 (58.7)	2905 (59.9)	158 (55.1)	38 (62.5)	
Age (n = 11,489)	19–29	709 (12.3)	561 (11.6)	17 (6.4)	3 (6.2)	<0.001
	30–39	1080 (17.1)	698 (13.7)	19 (7.7)	6 (7.2)	
	40–49	1214 (19.2)	832 (16.4)	37 (13.5)	4 (2.2)	
	50–59	1118 (19.0)	952 (20.1)	55 (18.5)	10 (19.4)	
	≥60	2045 (32.5)	1931 (38.2)	160 (53.8)	38 (65.0)	
House income (n = 11,468)	Lower	894 (14.3)	1226 (24.3)	199 (68.2)	46 (73.6)	<0.001
	Median	3125 (50.2)	2694 (53.8)	82 (30.4)	10 (18.8)	
	Upper	2137 (35.5)	1044 (21.9)	6 (1.4)	5 (7.6)	
Education	≤Primary school	1010 (16.1)	1216 (24.8)	137 (49.2)	31 (55.5)	<0.001
(n = 11,003)	Middle school	509 (8.7)	535 (11.3)	38 (13.6)	3 (4.4)	
	High school	1879 (32.2)	1513 (33.4)	67 (24.1)	19 (34.2)	
	≥College	2535 (43.0)	1475 (30.5)	32 (13.1)	4 (5.9)	
Diabetes (n = 11,489)	Absence	5606 (91.5)	4445 (89.8)	247 (85.8)	44 (72.6)	<0.001
	Presence	560 (8.5)	529 (10.2)	41 (14.2)	17 (27.4)	
BMI	Underweight	232 (3.9)	204 (4.3)	9 (3.3)	6 (8.1)	0.211
(n = 11,206)	Normal	2334 (39.6)	1837 (38.4)	99 (35.5)	21 (37.6)	
	Overweight	1388 (23.4)	1073 (21.8)	55 (21.4)	8 (15.3)	
	Obesity	2062 (33.1)	1737 (35.4)	118 (39.7)	23 (38.9)	
Smoking (n = 13,061)	Non-smoker	5167 (84.6)	4110 (83.9)	205 (72.9)	43 (71.2)	<0.001
	Smoker	950 (15.4)	820 (16.1)	80 (27.1)	18 (28.8)	
Alcohol drinking (n = 11,398)	Non-drinker	2745 (44.9)	2498 (50.9)	172 (58.7)	37 (58.2)	<0.001
	Drinker	3372 (55.1)	2437 (49.1)	113 (41.3)	24 (41.8)	
Tooth brushing/day	≤1	458 (7.0)	511 (10.3)	50 (19.3)	12 (19.0)	<0.001
(n = 11,119)	2	2246 (37.2)	1985 (41.3)	116 (41.7)	28 (55.5)	
	≥3	3304 (55.8)	2296 (48.4)	98 (39.0)	15 (25.5)	

* Weighted %; ** *p*–value was calculated by a complex sample chi-square test. F1 = secure with various food, F2 = secure without various food, F3 = sometimes insecure, F4 = often insecure.

**Table 4:** Association between food security and number of teeth. Results of multiple multinomial logistic regression models.

Food Security	21–28 versus 0–15		21–28 versus 16–20	
	Model 1	Model 2	Model 1	Model 2
F4 = Often insecure	5.52 *** (2.61–11.66)	1.79 (0.65–4.89)	5.36 *** (2.30–12.47)	3.80 ** (1.56–9.21)
F3 = Sometimes insecure	3.78 *** (2.65–5.40)	1.34 (0.81–2.21)	2.85 *** (1.83–4.44)	1.44 (0.85–2.43)
F2 = Secure without various food	1.38 *** (1.20–1.59)	0.92 (0.76–1.10)	1.24 * (1.04–1.48)	0.99 (0.80–1.22)
F1 = Secure with various food	Reference	Reference	Reference	Reference

Data are presented as OR (95% CI). OR: odds ratio; CI: confidence interval, * *p* < 0.05, ** *p* < 0.01 and *** *p* < 0.001. Model 1 unadjusted model. Model 2 adjusted for socioeconomic variables (sex, age, and household income, education), medical variables (diabetes mellitus, BMI) and behavior variables (smoking, alcohol drinking, tooth brushing).

## Data Availability

The data from the KNHANES VII survey can be accessed and downloaded from the KNHANES homepage (URL: https://knhanes.kdca.go.kr/knhanes/eng/index.do, accessed on 7 December 2022).

## References

[1] Marcenes W., Kassebaum N.J., Bernabé E., Flaxman A., Naghavi M., Lopez A., Murray C.J.L. (2013). Global burden of oral conditions in 1990–2010: A systematic analysis. J. Dent. Res..

[2] Pihlstrom B.L., Michalowicz B.S., Johnson N.W. (2005). Periodontal disease. Lancet.

[3] Orlandi M., Suvan J., Petrie A., Donos N., Masi S., Hingorani A., Deanfield J., D’Aiuto F. (2014). Association between periodontal disease and its treatment, flow-mediated dilatation and carotid intima-media thickness: A systematic review and meta-analysis. Atherosclerosis.

[4] Papapanou P.N., Susin C. (2017). Periodontitis epidemiology: Is periodontitis under-recognized, over-diagnosed, or both?. Periodontol. 2000.

[5] Struzycka I. (2014). The oral microbiome in dental caries. Pol. J. Microbiol..

[6] Han D.H., Khang Y.H., Lee H.J. (2015). Association between adult height and tooth loss in a representative sample of Koreans. Commun. Dent. Oral Epidemiol..

[7] Wennström A., Ahlqwist M., Stenman U., Björkelund C., Hakeberg M. (2013). Trends in tooth loss in relation to socio-economic status among Swedish women, aged 38 and 50 years: Repeated cross-sectional surveys 1968–2004. BMC Oral Health.

[8] Arora M., Schwarz E., Sivaneswaran S., Banks E. (2010). Cigarette smoking and tooth loss in a cohort of older Australians: The 45 and up study. J. Am. Dent. Assoc..

[9] Gondivkar S.M., Gadbail A.R., Gondivkar R.S., Sarode S.C., Sarode G.S., Patil S., Awan K.H. (2019). Nutrition and oral health. Dis. Mon..

[10] Shin H.S. (2020). The number of teeth is associated with diet quality in Korean adult population. Arch. Oral Biol..

[11] Maxwell A., Frankenberger T.R. (1992). Household Food Security: Concepts, Indicators, Measurements.

[12] Laraia B.A. (2013). Food insecurity and chronic disease. Adv. Nutr..

[13] Choi S.K., Fram M.S., Frongillo E.A. (2017). Very Low Food Security in US Households Is Predicted by Complex Patterns of Health, Economics, and Service Participation. J. Nutr..

[14] Bhaumik D., Wright C.D., Marshall T.A., Neiswanger K., McNeil D.W., Jones A.D., Shaffer J.R., Marazita M.L., Foxman B. (2023). Food insecurity and consumption of cariogenic foods in mothers and their two-year-old children in Appalachia. J. Public Health Dent..

[15] Lee M.H., Park J.W., Kwon Y.J. (2020). Household food insecurity and dental caries in Korean adults. Commun. Dent. Oral Epidemiol..

[16] Frazão P., Benicio M.H.D., Narvai P.C., Cardoso M.A. (2014). Food insecurity and dental caries in schoolchildren: A cross-sectional survey in the western Brazilian Amazon. Eur. J. Oral Sci..

[17] Park H.E., Song H.Y., Han K.D., Cho K.H., Kim Y.H. (2019). Number of remaining teeth and health-related quality of life: The Korean National Health and Nutrition Examination Survey 2010–2012. Health Qual. Life Outcomes.

[18] Kim S.W., Han K.D., Kim S.Y., Park C.K., Rhee C.K., Yoon H.K. (2015). The relationship between the number of natural teeth and airflow obstruction: A cross-sectional study using data from the Korean National Health and Nutrition Examination Survey. Int. J. Chron. Obstruct. Pulmon. Dis..

[19] WHO (2006). Definition and Diagnosis of Diabetes Mellitus and Intermediate Hyperglycemia: Report of a WHO/IDF Consultation.

[20] Park S.W., Kim B.G., Kim J.W., Park J.W., Kim J.I. (2018). A cross-sectional study on the pulmonary function of residents in two urban areas with different PM 10 concentrations: Data from the fourth Korea national health and nutrition examination survey (KNHANES) 2007–2009. Ann. Occup. Environ. Med..

[21] Schoenborn C.A., Adams P.F., Peregoy J.A. (2013). Health behaviors of adults: United States, 2008–2010. Vital Health Stat..

[22] Kim S.O., Bae E.M., Lee Y.N., Son J.S. (2021). Association between Consumption of Sugar-Sweetened Beverages and Risk of Cardiovascular Disease in Korean Men: Analysis Based on the Korea National Health and Nutrition Examination Survey 2014–2016. Korean J. Fam. Med..

[23] Mobley C., Marshall T.A., Milgrom P., Coldwell S.E. (2009). The contribution of dietary factors to dental caries and disparities in caries. Acad. Pediatr..

[24] Wiener R.C., Sambamoorthi U., Shen C., Alwhaibi M., Findley P. (2018). Food Security and Unmet Dental Care Needs in Adults in the United States. J. Dent. Hyg..

[25] Bruening M., MacLehose R., Loth K., Story M., Neumark-Sztainer D. (2012). Feeding a family in a recession: Food insecurity among Minnesota parents. Am. J. Public Health.

[26] Muirhead V., Quiñonez C., Figueiredo R., Locker D. (2009). Oral health disparities and food insecurity in working poor Canadians. Commun. Dent. Oral Epidemiol..

[27] Evans E.W., Hayes C., Palmer C.A., Bermudez O.I., Cohen S.A., Must A. (2013). Dietary intake and severe early childhood caries in low-income, young children. J. Acad. Nutr. Diet..

[28] Vedovato G.M., Surkan P.J., Jones-Smith J., Steeves E.A., Han E., Trude A.C., Kharmats A.Y., Gittelsohn J. (2016). Food insecurity, overweight and obesity among low-income African-American families in Baltimore City: Associations with food-related perceptions. Public Health Nutr..

[29] Seligman H.K., Jacobs E.A., López A., Tschann J., Fernandez A. (2012). Food insecurity and glycemic control among low-income patients with type 2 diabetes. Diabetes Care.

[30] Silverman J., Krieger J., Kiefer M., Hebert P., Robinson J., Nelson K. (2015). The Relationship Between Food Insecurity and Depression, Diabetes Distress and Medication Adherence Among Low-Income Patients with Poorly-Controlled Diabetes. J. Gen. Intern. Med..

[31] Marcos A., Nova E., Montero A. (2003). Changes in the immune system are conditioned by nutrition. Eur. J. Clin. Nutr..

[32] Hwang S.Y., Park J.E. (2022). The Relationship Between Periodontal Disease and Nutrient Intake in Korean Adults: The Korea National Health and Nutrition Examination Survey (KNHANES VII) from 2016–2018. Oral Health Prev. Dent..

[33] Hung H.C., Willett W., Ascherio A., Rosner B.A., Rimm E., Joshipura K.J. (2003). Tooth loss and dietary intake. J. Am. Dent. Assoc..

[34] Kim K.R., Hong S.A., Kwon S.O., Oh S.Y. (2011). Development of Food Security Measures for Korean National Health and Nutrition Examination Survey. Korean J. Nutr..

